# A Narrative Review of Endoprosthetic Reconstruction for Post-Traumatic Segmental Bone Defects After Fracture-Related Infection

**DOI:** 10.3390/antibiotics15100974

**Published:** 2026-10-01

**Authors:** Roshan Latifi, Yasaman Tavakoli, Fatemeh Kanaani Nejad, Mohammad Poursalehian, Jad Ismail, Robert A. Sershon

**Affiliations:** 1 Department of Orthopaedic Surgery, Aalborg University Hospital, 9000 Aalborg, Denmark; roshan.latifi@me.com; 2 Department of Medicine, Mazandaran University of Medical Sciences, Sari 4815733971, Iran; y.tavakoli@mazums.ac.ir; 3 Medical Imaging Research Center, Shiraz University of Medical Sciences, Shiraz 7193635899, Iran; fkanaani@sums.ac.ir; 4 Anderson Orthopaedic Research Institute, Alexandria, VA 22306, USA; jismail@aori.org (J.I.); rsershon@andersonclinic.com (R.A.S.)

**Keywords:** fracture-related infection, segmental bone defect, megaprosthesis arthroplasty, endoprosthesis, local antibiotic delivery, two-stage revision, silver coating, limb salvage

## Abstract

Segmental bone loss after fracture-related infection (FRI) may require biological reconstruction, endoprosthetic replacement, or amputation. This narrative review examines antimicrobial management, surgical selection, and outcomes of megaprosthetic reconstruction, with emphasis on adult lower-limb periarticular defects. Evidence is distinguished as direct FRI evidence, mixed clinical evidence, or indirect evidence from oncological reconstruction and periprosthetic joint infection (PJI). Megaprostheses provide immediate structural stability and may facilitate early weight-bearing in selected patients with extensive joint involvement, limited capacity for prolonged rehabilitation, or failed biological reconstruction. However, infection, soft-tissue failure, and mechanical complications remain substantial concerns. The frequently cited PJI incidence of 15–43% comes from heterogeneous megaprosthesis populations and is not an estimate specific to reconstruction after post-traumatic FRI. Small FRI series report encouraging short-term infection control, whereas mixed and megaprosthesis PJI cohorts demonstrate recurrent infection and further salvage surgery. Staged reconstruction is commonly reported, but comparisons between surgical strategies are vulnerable to selection bias, small samples, and inconsistent outcome definitions. Biological reconstruction outcomes provide contextual benchmarks rather than evidence of comparative superiority. Overall, megaprosthetic reconstruction is a reasonable salvage option when the expected benefit of immediate stability and preservation of joint motion outweighs the risks of infection and later implant failure. Treatment should integrate adequate debridement, appropriate systemic and local antimicrobial therapy, viable soft-tissue coverage, host optimization, and shared decision-making; the available evidence does not establish a universally preferred reconstruction algorithm.

## 1. Scope and Review Methods

This narrative review addresses adult segmental bone defects after FRI, principally around the knee and proximal femur. It examines how infection management, local antibiotic delivery, soft-tissue status, and host factors inform selection of megaprosthetic reconstruction. The evidence is concentrated in distal femoral, proximal tibial, and proximal femoral reconstruction; upper-extremity applications are discussed briefly as indirect context. Biological reconstruction is included to describe alternative treatment burdens and outcomes, rather than to imply a direct comparison with endoprosthesis. Reports involving established megaprosthesis PJI, oncological resection, or aseptic reconstruction are identified explicitly because their populations differ from patients undergoing initial reconstruction after FRI [1,2].

### 1.1. Literature Identification and Narrative Synthesis

PubMed was used for literature identification, and R.L. and Y.T. selected articles for the original narrative review. During revision, a supplementary PubMed search was performed on 22 September 2026 using the query reproduced in Appendix A, with a publication-date cutoff of that date. The supplementary search and targeted checks of cited studies informed the revision; they were not a prospectively registered systematic review. The synthesis focuses on adult FRI and segmental reconstruction, using consensus statements for infection-management principles, clinical reconstruction studies for outcomes, and laboratory or animal studies only to describe emerging technologies. Pediatric and nonorthopaedic reports were outside the clinical scope. Oncological and PJI reports were retained where they addressed a relevant technical or infection-management question and were labeled as indirect evidence.The manuscript is in accordance with the Scale for the Assessment of Narrative Review Articles (SANRA) [3].

### 1.2. Evidence Applicability

Direct FRI evidence comprises post-traumatic infected-defect cohorts or separately reported FRI subgroups. Mixed evidence combines FRI with PJI or other indications when a whole-cohort result is given. Indirect clinical evidence comprises established megaprosthesis PJI, oncological reconstruction, conventional arthroplasty, or aseptic trauma cohorts. Consensus recommendations and preclinical findings are identified separately. These categories describe applicability, not a formal certainty grade: a small uncontrolled FRI series remains at high risk of bias even though its population is directly relevant.

## 2. Background: Fracture-Related Infection and Segmental Bone Loss

### 2.1. Consensus Definition and Diagnosis

The contemporary framework rests on the 2018 AO/EBJIS consensus definition of FRI, developed through systematic review, an international survey of more than 2000 surgeons, and expert consensus, stratifying diagnosis into confirmatory and suggestive criteria [4]. The confirmatory criteria are a sinus/fistula/wound breakdown communicating with bone or implant; purulent drainage or intraoperative pus; phenotypically indistinguishable pathogens on ≥2 deep-tissue specimens; and microorganisms in deep tissue confirmed on histopathology [4]. The 2020 update added a fifth confirmatory criterion—>5 neutrophils per high-power field on histology in chronic/late presentations—and refined the suggestive criteria (new/persistent drainage, local inflammation, raised inflammatory markers, and suggestive imaging) [5,6]. In a multicenter validation of 637 patients, the presence of ≥1 confirmatory criterion achieved a sensitivity of 97.5% and specificity of 100% (AUROC 0.99), and the consensus definition captured 98.9% of infections versus only 49.8% for CDC organ/space SSI criteria [7,8]. Serum inflammatory markers are diagnostically weak in isolation (CRP sensitivity 67%, specificity 61%; WBC sensitivity 17%, specificity 95%) and should remain suggestive rather than confirmatory [9].

### 2.2. Microbiology Relevant to Reconstruction

*Staphylococcus aureus* and coagulase-negative staphylococci are common causes of FRI [10]. A retrospective comparison found a greater proportion of Gram-negative and multidrug-resistant isolates in FRI than in PJI, but these frequencies are setting-dependent and should not replace local microbiological data [11]. Polymicrobial infection is also relevant after open fractures [12]. In an indirect knee-PJI reconstruction cohort, polymicrobial infection was associated with additional debridement before endoprosthetic reconstruction (odds ratio 12.0) [13]. Sampling at least five deep-tissue specimens improves microbiological assessment; separate instruments should be used, and antimicrobial therapy should be adapted to the identified organisms [14,15,16].

### 2.3. Defining and Classifying the Segmental Defect

A critical-size bone defect is unlikely to heal without reconstruction, but a single length threshold cannot define this across anatomical sites and clinical settings [17,18]. In open tibial fractures, a 25 mm gap predicted nonunion, whereas almost half of defects meeting a smaller 1 cm and greater-than-50%-cortical-diameter definition healed without further surgery [19,20]. These findings are specific to their fracture populations. The Tetsworth–Glatt classification grades incomplete, minor, and segmental defects and provides a descriptive language for defect severity [21]. The Cierny–Mader system additionally considers the anatomical extent of infection and host physiology [22,23]. A systematic review of 1530 patients with FRI and critical-size defects reported infection cure of 83% after initial treatment and 94% at final follow-up, with 8% recurrence and 3% amputation [24]. These pooled results covered heterogeneous reconstruction strategies and do not establish an endoprosthesis-specific outcome or a preferred defect-size threshold.

## 3. Antimicrobial Management and Reconstruction

Infection management, mechanical stability, and viable soft-tissue coverage are interdependent requirements. Antimicrobial treatment should therefore be described in relation to the surgical source control achieved and the hardware remaining in situ [15,16].

### 3.1. Systemic Antimicrobial Principles

Fracture-related infection consensus recommendations support obtaining deep specimens before empirical antibiotics in clinically stable patients, followed by prompt culture-directed treatment [15,16]. After complete implant removal from a healed fracture and adequate debridement, the consensus suggests approximately six weeks of systemic treatment, with an oral switch when a suitable agent is available. With retained fixation, approximately 12 weeks of biofilm-active treatment is suggested when feasible; this is expert guidance, not a regimen validated specifically for megaprosthetic reconstruction. For susceptible staphylococci with retained hardware, rifampicin should be combined with another active agent and introduced after the wound has sealed and drains have been removed. During an implant-free interval in a staged FRI exchange, six weeks is suggested, but the optimal subsequent course is uncertain [16].

Broader osteomyelitis guidance supports no more than six weeks for adequately controlled infection without a retained implant; shorter courses after extensive resection remain a separate, incompletely defined setting [25]. These recommendations should not be transferred unchanged to an antibiotic spacer or definitive megaprosthesis. In the POvIV trial of 233 patients with FRI, oral treatment met the noninferiority criterion for reoperation in modified intention-to-treat analyses, but not in the prespecified adjusted per-protocol analysis [26]. This supports considering suitable oral agents in selected FRI patients; it does not determine treatment duration or establish equivalence for megaprosthesis PJI. Therapy after definitive endoprosthetic implantation requires individualized infectious-disease input, taking account of residual infection, cultures, susceptibility, implant retention, and toxicity [15,16].

### 3.2. Local Antibiotic Delivery and Dead-Space Management

Local antibiotic carriers can deliver antibiotics to a debrided cavity and help manage dead space. They supplement debridement, stabilization, systemic therapy, and soft-tissue reconstruction; laboratory elution or antibacterial activity alone does not establish clinical infection eradication [15,27].

Polymethylmethacrylate (PMMA) can be loaded with compatible antibiotics and fashioned into beads or structural spacers. As a nonresorbable carrier, it generally requires a plan for removal or exchange in staged reconstruction [28,29].

Antibiotic release from PMMA varies with cement formulation, antibiotic loading, geometry, and the surrounding environment. Laboratory and mixed laboratory–clinical studies describe an early release peak followed by lower release, with substantial residual antibiotic retained in the cement [30,31]. Differences between beads, spacers, and cement formulations, and experimental findings for cefazolin-loaded cement, should therefore be interpreted as formulation-dependent observations rather than universal clinical performance rankings [32,33,34].

Resorbable calcium sulfate can produce high local antibiotic concentrations without planned carrier removal. A clinical pharmacokinetic study of vancomycin-loaded calcium sulfate reported prolonged local exposure and generally low systemic levels, but this does not establish safety for every dose, host, or renal-function category [35]. Evidence for calcium sulfate/hydroxyapatite composites includes an animal implant-associated infection model; claims about the optimal clinical timing or type of FRI defect remain uncertain [28,36]. Antibiotic-loaded β-tricalcium phosphate/calcium sulfate and borate bioactive glass have demonstrated antibiofilm or therapeutic effects in laboratory and animal studies, respectively, rather than definitive efficacy in human FRI reconstruction [37,38].

Coatings require a similar distinction between prevention and treatment. A gentamicin-coated tibial nail cohort addressed prevention after open fracture, whereas infected-nonunion series examined coated nails as part of an infection-treatment strategy [39,40]. A network meta-analysis of coating studies suggested reduced implant-associated infection, but heterogeneous populations and indirect comparisons limited technology rankings for FRI salvage [41]. Continuous local antibiotic perfusion has also been evaluated in a retrospective FRI cohort; its reported associations require prospective confirmation [42]. Table 1 separates evidence type and clinical purpose for representative carriers and coatings.

## 4. Endoprosthetic Reconstruction and Patient Selection

### 4.1. From Oncology to Septic Trauma

A systematic review of 2598 non-oncological megaprostheses found that distal and proximal femoral replacements accounted for most reconstructions [1]. These data include periprosthetic fracture and aseptic failure and therefore describe the wider use of megaprostheses rather than outcomes specific to FRI. After septic trauma, reconstruction is additionally affected by previous operations, scarred soft tissues, bone loss, and the demands of long-term implant survival [45].

### 4.2. Factors Supporting Consideration of Megaprosthesis

Megaprosthetic reconstruction may be considered for extensive periarticular loss, inadequate remaining bone for conventional reconstruction, failed biological reconstruction, or a patient unable to tolerate prolonged frame treatment or repeated grafting [44,45,46,47,48,49]. Defect length contributes to this assessment, but reported associations between length and biological reconstruction outcomes do not establish a universal 6 cm cutoff. Immediate structural stability is an advantage; the timing of weight-bearing still depends on implant fixation, soft-tissue reconstruction, and the rehabilitation protocol [1,44].

### 4.3. Initial Reconstruction After FRI and Revision of an Infected Megaprosthesis

These are distinct clinical problems. Small direct FRI series and mixed FRI/PJI cohorts describe staged debridement, removal of infected fixation, dead-space management, and subsequent megaprosthetic reconstruction after reassessment of infection and soft tissues [2,44,50]. By contrast, comparisons of debridement, antibiotics, and implant retention (DAIR); single-stage exchange; and two-stage exchange mainly concern an already infected megaprosthesis. In one mixed oncological/non-oncological PJI cohort, infection-free implant survival was 5/7 (71.4%) after two-stage revision, 12/31 (38.7%) after DAIR, and 0/5 after single-stage revision [51]. These small, nonrandomized groups differ in treatment selection; the five single-stage cases cannot establish that this approach is uniformly ineffective. Oncological megaprosthesis data also associate complete component removal with better infection control than stem retention [52]. These observations support careful consideration of implant removal for chronic infection but do not prove superiority of a single protocol for initial reconstruction after FRI. Data from conventional arthroplasty trials, with different eligibility and soft-tissue conditions, remain indirect [53].

### 4.4. Interstage Treatment and Reimplantation Timing

Published protocols vary. An indirect knee-PJI endoprosthetic series used six weeks of intravenous antibiotics followed by at least six weeks of oral therapy, whereas the mixed FRI/PJI distal femoral series used culture-directed systemic treatment for four to six weeks and repeated the first stage when infection persisted [2,13]. An antibiotic spacer remains foreign material and is not equivalent to a definitive implant-free, healed-fracture setting. Reviews of conventional PJI have not established a benefit from a routine antibiotic-free interval [54]. Likewise, timing associations reported in hip-PJI cohorts should not define a universal 4–11-week reimplantation window for FRI or megaprosthesis reconstruction [55]. Reimplantation is individualized according to wound healing, soft-tissue coverage, clinical findings, microbiology, and the overall response to treatment; no single laboratory result proves eradication [15,16].

### 4.5. Technical Considerations and Extensor Mechanism Reconstruction

Restoration of length, rotation, and soft-tissue balance is important. Rotational planning findings from sarcoma reconstruction provide indirect technical guidance for septic trauma [45,56]. Direct post-traumatic evidence for proximal tibial extensor reconstruction is limited to small series. Calori et al. reported nine patients treated with tendon reconstruction, synthetic ligament augmentation, and gastrocnemius coverage. One reconstructed patellar tendon ruptured after a subsequent traumatic event and required revision (1/9, 11.1%); no quadriceps failure or patellar fracture was reported. The mean WOMAC score was 74.8 at 18 months, and no infection recurrence was observed during a mean 18-month follow-up [50]. Absence of reported tendon rupture in the other eight patients is not a validated overall functional success rate. Evidence on stem fixation and instability prevention largely comes from mixed fracture or non-oncological cohorts and must be interpreted in that context [57,58].

### 4.6. Soft-Tissue Coverage as an Infection-Control Variable

Soft-tissue coverage can determine whether reconstruction is feasible. The PARITY flap analysis concerns lower-extremity bone tumors and is indirect evidence for FRI; its association with fewer reoperations does not establish a causal benefit in septic trauma [59]. Gastrocnemius and free-flap reconstructions have been reported in post-traumatic proximal tibial reconstruction and infected oncological or knee-PJI settings [50,60,61,62,63,64,65,66]. In direct tibial FRI evidence, a staged protocol for combined bone and soft-tissue loss achieved 93.5% limb salvage with infection eradication in salvaged limbs, but it was not a comparison of megaprosthesis with biological reconstruction [67]. These populations support the need for an orthoplastic plan while providing different estimates of reconstructive outcome.

### 4.7. Antibacterial-Coated Megaprostheses

The principal mixed FRI/PJI distal femoral cohort used silver-coated implants, but lacked an uncoated comparison and therefore could not isolate a coating effect [2]. Other studies concern high-risk oncological reconstruction or established end-stage PJI, with inconsistent findings [68,69]. A meta-analysis also reported uncertainty around the effect of silver coating [70]. Similarly, the small direct FRI Compress series combined an antibacterial hydrogel with multiple elements of staged care; its favorable infection outcome cannot be attributed to the coating alone [44]. Silver exposure and local argyria have been described, supporting surveillance rather than an assumption of universal absence of toxicity [71,72]. The HIPrevision trial addresses antimicrobial-coated revision hip prostheses and may inform future practice, although its population differs from initial post-traumatic megaprosthetic reconstruction [73].

### 4.8. Upper-Extremity Evidence and Limits of Extrapolation

Upper-extremity data are substantially less extensive and derive mainly from oncological reconstruction, severe proximal humeral bone loss, and shoulder PJI [74,75,76,77,78]. Reverse megaprosthetic constructs may restore an articulation in selected cases, but reported complications and reoperations are frequent [76]. Shoulder spacer and allograft–prosthesis composite series provide technical context rather than a reliable post-traumatic FRI-control estimate [77,78]. Deltoid function, nerve integrity, and reconstructable soft tissues are central considerations. Lower-limb outcome ranges and weight-bearing arguments should not be transferred directly to the upper limb.

## 5. Outcomes and Complications

### 5.1. Infection Eradication and Reinfection

Infection outcomes depend on both the original indication and the definition of failure. Direct FRI reconstruction series should be separated from studies treating a subsequent megaprosthesis PJI. The frequently quoted 15–43% incidence range comes from heterogeneous megaprosthesis indications and is not an FRI-specific estimate [51]. In a non-oncological cohort of 219 megaprostheses, 38 developed PJI (17.4%), while treatment outcomes were analyzed in 33 patients; two-year survival free of the study-defined treatment failure was 65.1%, and six of the 33 underwent amputation [79]. Another mixed oncological/non-oncological PJI cohort reported infection-free implant survival of 42% [51]. A separate megaprosthesis cohort reported amputation in 37% of infected patients, a result that should not be generalized to all patients reconstructed after FRI [80]. Long-term recurrence after revision of oncological megaprosthesis PJI also reflects a different population [81]. Table 2 presents denominators, clinical settings, and study-specific endpoints; its percentages should not be ranked or pooled as if they measured the same outcome.

### 5.2. Function and Survivorship

Functional outcomes also reflect the underlying indication. A mixed proximal femoral cohort reported lower Musculoskeletal Tumor Society (MSTS) scores in non-oncological than oncological patients [82]; this is not a direct comparison of FRI treatments. The knee-PJI spacer series reported a mean MSTS score of 77% and 100° knee range of motion, whereas the direct FRI Compress series reported a mean Lower Extremity Functional Scale score of 52.5% [13,44]. These measures cannot be compared as equivalent outcomes. Long-term complication-free survival data for non-oncological proximal femoral replacement include substantial instability and revision burdens [58]. The Henderson classification can organize soft-tissue, loosening, structural, and infection-related failures, although its tumor-progression category is inapplicable to FRI [83].

### 5.3. Risk Factors for Failure

Potential risk factors differ by population. Greater resection length was associated with recurrence in the mixed distal femoral FRI/PJI cohort [2]. Associations involving body mass index, difficult-to-treat or polymicrobial organisms, and previous surgery arise largely from PJI or oncological megaprosthesis studies [81,84,85,86]. They inform clinical assessment but do not constitute a validated prediction model for initial reconstruction after FRI.

## 6. Biological Reconstruction as Contextual Evidence

No randomized trial directly compares megaprosthesis with biological reconstruction in FRI. Published outcomes therefore provide contextual benchmarks, with substantial differences in defect location, joint involvement, host status, and endpoint definitions [24]. Reviews of induced-membrane reconstruction report substantial rates of union and infection control, sometimes requiring repeat grafting [87,88]. Tibial bone-transport series report high union and infection-control rates but also prolonged treatment and docking-site procedures [89,90,91]. A meta-analysis comparing distraction osteogenesis with induced membrane did not find a statistically significant difference in pooled union or infection-elimination rates, but heterogeneity and limited comparative data restrict inference [92]. Vascularized fibular grafting can reconstruct long defects, with donor-site morbidity and graft-related complications; combined approaches have been described for extensive infected defects [93,94,95]. A small randomized comparison of two biological techniques does not answer the endoprosthesis-versus-biological-reconstruction question [96]. The reamer–irrigator–aspirator is a graft-harvest adjunct, rather than a standalone reconstruction [97,98].

Biological reconstruction may provide durable living bone after union and is often considered when the joint is salvageable, the host can tolerate rehabilitation, and adequate vascularity and coverage are available. Megaprosthesis may offer a shorter route to structural reconstruction and preservation of joint motion, while introducing continuing implant-related risks [47,99,100]. These are clinical trade-offs, not demonstrated comparative treatment effects. Table 3 is an illustrative decision framework. Its approximate size categories are pragmatic discussion aids, not validated thresholds. Selection also depends on periarticular involvement, residual bone stock, vascular and soft-tissue status, pathogen, host grade, previous procedures, life expectancy, rehabilitation capacity, and patient preference.

## 7. Multidisciplinary Care and Consideration of Amputation

The FRI Consensus Group advocates multidisciplinary management involving orthopaedic surgery, infectious diseases, microbiology, and plastic surgery, with rehabilitation and psychological support as needed [15]. An observational FRI study associated incomplete infectious-disease consultation with treatment failure, supporting continuity of specialist input without proving causality [102]. Glycemic control, nutrition, smoking, vascular disease, renal function, and immunosuppression should be addressed [103]. The McPherson system incorporates infection type, systemic host grade, and local extremity status and was developed for PJI [104]. Associations between host grade and repeat two-stage knee-PJI outcomes, and comparative assessments of PJI classifications, are therefore indirect prognostic evidence for post-traumatic reconstruction [105,106]. Complex cases benefit from coordinated assessment in experienced centers [15,45].

Amputation may be considered when infection, vascular compromise, soft-tissue loss, or the cumulative burden of reconstruction makes limb salvage unlikely to meet the patient’s goals. Studies of fracture-related infection identify renal disease, injury characteristics, pathogens, and repeated operations as relevant risk markers [107]. The American Academy of Orthopaedic Surgeons (AAOS) guideline on severe lower-extremity trauma advises against using extremity scoring systems alone to choose salvage or amputation; its scope differs from chronic FRI reconstruction [108]. Amputation also carries substantial morbidity. In a severe knee-PJI salvage cohort, 37.0% of patients undergoing above-knee amputation required further revision surgery, two-year mortality was 28.3%, and only 45.7% were fitted or intended to be fitted with a prosthesis [109]. These observational results reflect a selected PJI population and are neither causal comparisons nor FRI-specific risk estimates. Counseling should address recurrent infection, wound or stump complications, pain, prosthetic fitting, mobility, and the possibility of further surgery, alongside the burden of attempted reconstruction.

## 8. Limitations and Future Directions

The principal evidence gap is the absence of direct randomized comparisons of megaprosthesis with biological reconstruction or amputation after FRI. Available endoprosthetic series are small and retrospective; oncological, aseptic, and PJI populations are often combined, and those reaching definitive reimplantation may represent a selected subset [1,2,24]. Infection recurrence, chronic suppression, further surgery, implant survival, and amputation must be reported separately rather than compressed into an undefined success rate. Future prospective registries should stratify the original indication, infection status at implantation, host and soft-tissue factors, previous procedures, and outcomes from the first treatment stage.

### 8.1. Limitations of the Review

This is a narrative review with purposive selection rather than a comprehensive systematic search. Reliance on PubMed and selected references may omit relevant studies and introduce selection and publication bias. The supplementary search does not remove these limitations. Formal risk-of-bias assessment, certainty grading, and quantitative pooling were not performed. Heterogeneous indications, treatment selection, incomplete reporting, and variable follow-up limit comparison across series; some study details could only be assessed from abstracts. Lower-limb periarticular reconstruction dominates the evidence, limiting conclusions for upper-extremity and diaphyseal defects. Consequently, the proposed framework is an aid to discussion rather than a validated treatment guideline.

### 8.2. Research Priorities and Emerging Adjuncts

Prospective studies should distinguish initial megaprosthetic reconstruction after FRI from revision of an infected megaprosthesis and evaluate staging, interstage antibiotics, implant coatings, function, and long-term survivorship [53,54,70,73,110]. Studies of biological and endoprosthetic reconstruction should report case mix and treatment burden before making comparative claims. Regenerative carriers and custom porous implants require clinical evaluation in defined infection settings [37,38,111]. Placental-derived membranes have wound-healing and preclinical regenerative applications, but have not been validated for dead-space management or bone regeneration in established FRI [112,113,114]. Their potential role remains investigational and does not support replacing established soft-tissue coverage.

## 9. Conclusions

Megaprosthetic reconstruction is a reasonable salvage option for carefully selected adults with extensive periarticular bone loss after FRI, particularly when immediate structural stability and preservation of joint motion are priorities [1,44,45]. The decision requires adequate debridement, appropriate antimicrobial treatment, viable soft-tissue coverage, and assessment of host and rehabilitation capacity [15,16]. Staged reconstruction with removal of infected hardware is commonly supported by retrospective reports, but its comparative superiority for all post-traumatic defects has not been established. Biological reconstruction remains an important alternative when durable bone restoration is feasible. Reported infection and amputation rates from mixed megaprosthesis populations should inform counseling as indirect evidence, not precise FRI-specific predictions. Treatment selection and sequencing should remain individualized and shared with the patient.

## Figures and Tables

**Table 1:** Local antimicrobial carriers and coatings by evidence type and clinical purpose.

Carrier or Coating	Evidence Type and Setting	Clinical Purpose	Interpretation for FRI Reconstruction	Refs.
PMMA beads or spacers	Laboratory elution; clinical use and consensus	Adjunctive treatment; dead space and temporary stability	Release depends on formulation and geometry; nonresorbable carrier with planned management or removal. Elution results do not measure clinical eradication.	[27,28,29,30,31,32,33,34]
Calcium sulfate	Clinical pharmacokinetics in bone and soft-tissue infection	Adjunctive treatment	Resorbable carrier with high local exposure; pharmacokinetics do not prove superior cure or universal systemic safety.	[35]
Calcium sulfate/HA	Mouse implant-associated infection model; review	Experimental adjunctive treatment	Preclinical findings do not establish an optimal timing or defect indication in adult FRI.	[28,36]
β-TCP/calcium sulfate; borate bioactive glass	In vitro biofilm work; rabbit osteomyelitis model	Experimental prevention or treatment	Laboratory antibiofilm activity and animal treatment effects require separate interpretation from human outcome data.	[37,38]
Antibiotic-coated intramedullary nails	Open-fracture prevention cohort; review of infected-nonunion cohorts	Prevention [39]; treatment [40]	Primary infection prevention and treatment of established nonunion are different endpoints; neither is a controlled megaprosthesis comparison.	[39,40]
DAC hydrogel	Mixed coating studies; comparative hip-PJI series; small FRI series	Prevention of implant infection; adjunct at reimplantation	Coating effects cannot be separated from staged care in the small direct FRI series.	[41,43,44]

Abbreviations: DAC, defensive antibacterial coating; FRI, fracture-related infection; HA, hydroxyapatite; PJI, periprosthetic joint infection; PMMA, polymethylmethacrylate; β-TCP, beta-tricalcium phosphate. Clinical purpose refers to the cited evidence, not proven interchangeability of the products.

**Table 2:** Selected clinical series with population and outcome definitions retained.

Study and Population	Protocol and Previous Operations	Soft-Tissue Reconstruction	Study Endpoint and Result	Follow-Up and Amputation
Fiore 2023 [2] Mixed: 14 FRI and 31 PJI; distal femur; *n = 45*	Staged silver-coated replacement. Prior infection surgery in 28/45; 12/28 had >3 operations.	Flaps in 8/45 at first stage.	Recurrence included sinus, infection surgery, or chronic antibiotics. Recurrence-free survival of 91.1% at 2 years and 75.7% at 5 years. FRI subgroup: 64.3% at 5 years.	Mean 43 months. Amputation in 2/45 (4.4%) after reimplantation. Two pre-reimplantation amputations were excluded.
Corona 2021 [44] Direct: infected post-traumatic distal femoral defects; *n* = 10 (11 implants)	Two-stage Compress implant plus DAC. Mean of 4.4 previous operations.	One patient required an anterolateral thigh free flap for skin necrosis (1/10, 10%).	Infection eradication: 10/10 (100%). Defined by a healed wound, no recurrence with the same organism at one year, no subsequent surgery for infection, no PJI-related mortality, and no suppressive antibiotic therapy. Two aseptic loosenings required femoral-component revision.	Median 27 months (12–50). All limbs salvaged.
Ippolito 2021 [13] Indirect: revision knee arthroplasty or endoprosthetic PJI; *n* = 21	Two-stage treatment using intramedullary rod-stabilized antibiotic spacers followed by endoprosthetic reconstruction. Mean operations before infection: 4.1 ± 3.0.	Flap coverage in 12/19 patients retaining a limb and endoprosthesis (63%): 9 gastrocnemius, 2 free, and 1 fasciocutaneous flap.	Author-reported infection eradication: 18/21 (86%). A formal definition of antibiotic-free eradication was not specified. Chronic oral antibiotic therapy was reported in 3 patients; 2 late reinfections were reported.	Mean follow-up: 10.3 ± 6.4 years (range 1.7–24.3), as reported in the Results. Above-knee amputation: 1/21 (4.8%) following persistent infection despite repeated debridement and spacer exchange.
Schlossmacher 2025 [51] Indirect: oncological/non-oncological megaprosthesis PJI; *n* = 50	DAIR, single-, two-, or multistage revision. Median of 4 prior operations. Initial indication: PJI in 19, loosening in 13, oncology in 11, and fracture in 7.	NR	Infection-free implant survival in 21/50 (42%); healed wound without fistula, drainage, pain, or recurrence. Suppression and PJI-related death were recorded separately.	Mean of 19 months. Amputation 7/50 (14%).
Sukhonthamarn 2021 [79] Indirect: non-oncological megaprosthesis PJI; 38/219 developed PJI, 33 analyzed	Mainly DAIR (27/33); other procedures included exchange and resection. Original indications included prior PJI, fracture, and loosening. Prior-operation count NR.	NR	65.1% survival free of study-defined treatment failure at 2 years; unplanned revision/salvage. Further outcomes included suppression, arthrodesis, resection, and amputation.	Mean of 36 months. Amputation in 6/33 (18.2%); mortality in 5/33 (15.2%).
Calori 2016 [50] Direct: post-traumatic septic proximal tibial defects; *n* = 9	Two-stage treatment: hardware removal, resection of infected bone/non-union, and antibiotic spacer placement, followed by cemented proximal tibial megaprosthesis implantation after a mean interval of 3 months. Number of previous operations: NR.	Medial gastrocnemius flap for extensor mechanism reconstruction in 9/9 patients (100%), also providing skin coverage in 5/9 (56%). Tendon-plasty and LARS synthetic ligament reinforcement were used in all patients.	No infection recurrence reported in 9/9 patients. A formal definition of infection eradication and suppressive antibiotic use were not reported. One traumatic rupture of the reconstructed patellar tendon required revision surgery at 18 months; no revision for prosthetic loosening or device failure was reported.	Mean follow-up: 18 months (range 9–36) after the second-stage procedure. Amputation: not explicitly reported.

All six studies are retrospective. Direct means post-traumatic septic reconstruction; mixed means FRI and PJI; indirect means established PJI or a different original indication. NR means not reported in the reviewed full text. Time-to-event estimates and crude proportions are not interchangeable. For abstract-only reports, the handling of chronic suppression, reoperation, implant removal, and amputation within the infection endpoint could not be fully established. Abbreviations: DAC, defensive antibacterial coating; DAIR, debridement, antibiotics, and implant retention; FRI, fracture-related infection; PJI, periprosthetic joint infection.

**Table 3:** Illustrative decision framework for reconstruction after FRI.

Illustrative Defect Context	Biological Options to Discuss	Factors Supporting Endoprosthesis Consideration	Refs.
Small contained or short segmental defect	Autograft, with stable fixation and adequate coverage	Joint destruction or failed reconstruction may matter more than length; otherwise replacement is less commonly considered.	[17,99]
Intermediate defect, roughly 5–6 cm	IMT or bone transport; graft choice depends on volume and vascularity	Periarticular involvement, limited bone stock, prior failure, or inability to tolerate prolonged reconstruction.	[46,47,99]
Long segmental defect, roughly 6–10 cm	IMT, transport, or VFG in suitable hosts	Extensive joint involvement and expected functional benefit, balanced against implant and infection risks.	[44,93,99]
Very long defect, roughly >10 cm	Transport or combined biological techniques, including IMT plus VFG	Feasible implant fixation and reliable soft-tissue coverage are necessary; poor coverage alone is not an indication for megaprosthesis.	[44,94,95,101]
Periarticular loss at any length	Joint-preserving reconstruction where feasible; arthrodesis in selected cases	Potential preservation of joint motion, with adequate extensor or abductor function and a realistic rehabilitation plan.	[44,45,50]

Size categories are approximate, pragmatic, and unvalidated; they do not prescribe a preferred treatment. Adequate infection management, stability, vascularity, and reconstructable soft tissues are required for either approach. Abbreviations: IMT, induced membrane technique; VFG, vascularized fibular graft.

## Data Availability

No new data were created or analyzed in this study. Data sharing is not applicable to this article.

## Abbreviations

The following abbreviations are used in this manuscript:

AUC	Area under the curve
CaSO_4_	Calcium sulfate
CKD	Chronic kidney disease
DAC	Defensive antibacterial coating
DAIR	Debridement, antibiotics, and implant retention
FRI	Fracture-related infection
IMT	Induced membrane technique
MRSA	Methicillin-resistant *Staphylococcus aureus*
MSTS	Musculoskeletal Tumor Society
PJI	Periprosthetic joint infection
PMMA	Polymethylmethacrylate
RIA	Reamer–irrigator–aspirator
VFG	Vascularized fibular graft
AAOS	American Academy of Orthopaedic Surgeons
HA	Hydroxyapatite
LEFS	Lower Extremity Functional Scale
SANRA	Scale for the Assessment of Narrative Review Articles
WOMAC	Western Ontario and McMaster Universities Osteoarthritis Index

## Appendix A. Supplementary PubMed Search

Search performed during revision on 22 September 2026. Database: PubMed, accessed through the NCBI E-utilities interface. Publication-date limits: 1 January 1900 to 22 September 2026. No language filter was applied to the query. The search returned 128 records; this is a retrieval count, not an included-study count. The query was used for supplementary narrative identification alongside targeted checks of cited publications and is not a reconstruction of the original search history.

(“fracture-related infection”[Title/Abstract] OR “post-traumatic”[Title/Abstract] OR posttraumatic[Title/Abstract] OR “infected nonunion”[Title/Abstract]) AND (megaprosthe*[Title/Abstract] OR endoprosthe*[Title/Abstract]).

Records unrelated to adult orthopaedic reconstruction or not relevant to the clinical questions were not used for the synthesis. Selected FRI reports were interpreted alongside clearly labeled consensus, PJI, oncological, and preclinical sources.
